# Comparative analysis of the prognosis of external beam radiation therapy (EBRT) and EBRT plus brachytherapy for glioblastoma multiforme: a SEER population-based study

**DOI:** 10.1186/s13014-022-02141-z

**Published:** 2022-10-28

**Authors:** Kai Yang, Yan Ma, Guo Chen, Shaojie Zeng, Ting Guo, Zelong Yang

**Affiliations:** 1 Department of Hepatobiliary Surgery, Xi Jing Hospital, Air Force Medical University, Xi’an, China; 2 Department of Gynecology and Obstetrics, Xi Jing Hospital, Air Force Medical University, Xi’an, China; 3 State Key Laboratory of Cancer Biology, Department of Pharmacogenomics, Air Force Medical University, Xi’an, China; 4 Department of Obstetrics, West China Second University Hospital, Sichuan University, Chengdu, China

**Keywords:** External beam radiation therapy, Brachytherapy, GBM, Competing risk model, Propensity score matching

## Abstract

**Objective:**

Radiotherapy is one of the effective ways to treat glioblastoma multiforme (GBM). We aimed to explore the prognostic difference between external beam radiotherapy (EBRT) and EBRT combined with brachytherapy (EBRT + BT).

**Methods:**

The GBM patients from the Surveillance, Epidemiology, and End Results (SEER) database were divided into two cohorts: the EBRT cohort and the EBRT + BT cohort. Kaplan–Meier (KM) analysis and Cox proportional hazards regression were used to determine the underlying risk factors for overall survival (OS) and disease-specific survival (DSS). And the competing risk model and propensity score matching (PSM) was adopted to eliminate potential biases. We also conducted subgroup analyses and interaction tests as well.

**Results:**

There was a total of 41,010 eligible GBM patients. The median OS (15 months) and DSS (17 months) of the EBRT + BT cohort were significantly longer than that of the EBRT cohort (OS = 11 months, DSS = 12 months). After using the competing risk model and PSM, we found that only advanced age was the independent risk factor, while only EBRT + BT was the independent protective factor (HR = 0.84, 95%CI [0.74,0.96], *p* = 0.01). EBRT had universal effects in the treatment of GBM, and EBRT + BT had a more pronounced protective effect in the subgroups of males (HR = 0.81, 95%CI [0.68,0.97], *p* = 0.02) and local excision (HR = 0.82, 95%CI [0.34,0.95], *p* = 0.01).

**Conclusions:**

The therapeutical effect of EBRT + BT treatment is better than that of EBRT alone, especially in male patients or patients who have undergone local resection. Our findings may provide novel evidence to develop a better radiotherapy strategy for GBM patients.

## Introduction

Gliomas are the most frequent intracranial nervous system tumor and involve diffuse or circumscribed patterns. It is classified from grade I to grade IV, and grade IV, glioblastoma multiforme (GBM), is the most malignant [1]. Although there are several therapeutical methods for GBM now, and tremendous cancer mechanisms have been explored, the prognosis of GBM patients remains poor; only about 5% of GBM patients can survive over five years [2]. Among GBM patients who have undergone complete tumor resection, more than 80% of patients will experience tumor recurrence, and the relapse lesions are usually located within 2 cm of the resection margin [3]. The high recurrence propensity contributes to the poor prognosis of GBM.

Postoperative concomitant external beam radiotherapy (EBRT) plus temozolomide has been recognized as the standard treatment for GBM [4]. The radiotherapy requires at least 55 Gy of EBRT to control the tumor. Still, if the radiation dose of EBRT is higher than 60 Gy, radiation-related necrosis would occur in the normal brain tissue and result in irreversible damage. So, the effect of EBRT is limited by the radiation dose [5]. Brachytherapy (BT) has also emerged as a promising treatment method for advanced or recurrent GBM. In particular, iodine 125 brachytherapy is regarded as a kind of salvage therapy and gained good clinical response [6]. Wernicke et al. found that using Cs-131 to BT to treat patients with recurrent GBM combined with bevacizumab can significantly alleviate radioactive tissue necrosis and inhibit tumor progression [7]. In a dilemma, whether the clinical efficacy of EBRT combined with BT (EBRT + BT) is better than that of EBRT alone is still inconclusive.

Competing death risks should be considered when evaluating the prognosis of cancer patients because tumor patients are more easily subjected to competitive death, such as suicide, cerebrovascular accident, and cardiac death [8, 9]. If the number of competitive events exceeds 10% of the overall events, the results of Cox regression will most likely be incorrect. Therefore, the competing risk model is more convincing and conducive to assessing the association between variables and GBM-specific deaths.

In this SEER-based study, we achieved "post-randomization" through PSM and explored the prognostic difference between the two irradiation methods based on competing risk analyses. To our knowledge, this is the first and the most extensive population-based study focused on the prognostic difference between the patients who received the two radiotherapy methods. We may have provided a basis for precise treatment and individual medicine through subgroup analyses and interaction tests.

## Materials and methods

### Data collection

SEER is the most authoritative and comprehensive cancer registry in the world, which includes patient records from 18 states in the U.S and might cover 36.7% population of Americans [10]. We recruited cancer cases and related data from the SEER database using SEER*Stat software (version 8.3.5). The data in the SEER program are publicly available, and our research conformed with the revised Declaration of Helsinki, so ethical consent is not required.

### Data selection

There were 144,820 participants diagnosed with GBM from 1975 to 2016 in the SEER database enrolled in the study. Next, a total of 41,010 eligible patients with GBM located in the brain (Site Code C71.0, C71.1, C71.2, C71.3, C71.4, C71.5, C71.6, C71.7, C71.8, C71.9, C72.0), correct ICD-O-3 code (Histological type 9440, 9441, 9442), histologically diagnosed GBM (Glioblastoma, Gliosarcoma, Giant cell glioblastoma), complete prognostic information (survival month > 0 months), underwent one of the treatment modalities (EBRT or EBRT + BT) were included.

Cases with primary GBM located outside the cerebrum, with ambiguous prognostic information, were excluded. Cases with non-GBM histological types or with no ICD-O-3 codes were excluded. Patients with radiation therapies other than EBRT or EBRT + BT were excluded, and one case with unknown history of malignancy was excluded.

### Variable conversion and definition

We collected the data of each as follows: age, gender, race, marital status, diagnosis year, chemotherapy history, surgery history, primary tumor site, tumor size, other malignance histories, pathological type, survival time, survival event (GBM-specific death and competitive risk death), and applied to subsequent statistical analysis. Age at GBM diagnosis was divided into four groups as a categorical variable: Age 50 and under, 51–60, 61–70, 71 and above. Racial classification refers to white people, black people, and other races. The marital variable was grouped into unmarried, married, divorced, and others. Similarly, the diagnosis year of GBM was categorified into the following intervals: 1975–2000, 2001–2005, 2006–2010, and 2011–2016. The chemotherapy history of GBM was a dichotomous variable, defined as receiving chemotherapy or not. The surgery history of GBM was categorized as no surgery, local excision, subtotal resection, gross total resection, and unknown history. Primary tumor sites of GBM were classified into five groups: frontal, temporal, parietal, occipital, and other areas in the brain. Pathological tumors were grouped into giant cell GBM (gcGBM), gliosarcoma, and GBM.

We included the history of malignant tumors as a binary variable. We converted the tumor size from a continuous variable to a categorical variable: ≤ 4 cm, > 4 cm, unknown length. There were three classifications for the survival event variable: disease-specific death, competitive death, and survival. OS and DSS were used as the survival time variables.

### Statistical methods

Statistic software R (version 4.1.3, https://www.r-project.org/) was used for analysis in this study. Kaplan–Meier survival analysis, Log-rank test, and univariate and multivariate Cox regression were conducted by the “survival” package. There are three kinds of events: survival, GBM-specific death, and non-GBM-specific death. Non-GBM-specific death is a competing event of GBM-specific death; in the context of competing events, traditional statistical approaches are less calibrated because it can’t be assumed whether GBM-specific death occurs if the subjects are followed up long enough. So, we selected GBM-specific death as the outcome of interest, whereas non-GBM-specific death was considered a competing risk event and a patient alive was regarded as a censored event. We calculated the cumulative risks for categorical variables, especially EBRT and EBRT + BT, using the cumulative incidence function (CIF) of the “cmprsk” package [11] in R; Gray’s test was used to identify the significant difference among groups. The “forestplot” package plotted the Nelson-Aalen cumulative hazard curves in R to visualize the cumulative risk difference. In the multivariate analysis of competing risk regression, we performed Fine & Gray proportional subdistribution hazard model [12] to recognize the independent risk factors by the “cmprsk” package. PSM was adopted to reduce the selection bias of the two groups of baseline variables, including age, sex, race, marital status, year of diagnosis, histological type, surgery method, chemotherapy, tumor size, tumor history, and primary site. Logit model was used to calculate propensity scores. The match ratio of PSM is 2:1 (EBRT: EBRT + BT); the nearest neighbor matching approach was selected; the caliper value was set as 0.02. The “MatchIt” package conducted PSM in R [13]. The clinicopathological features of the patients were reevaluated. SMD (Standardized Mean Difference) < 0.1, *p* < 0.05, and the density map were used to prove the baseline balance after PSM. The subgroup analysis and interaction test were conducted after PSM. All statistical tests were two-sided, and *p* < 0.05 were considered statistically significant.

## Result

### Demographic and clinical characteristics

**Fig. 1:**
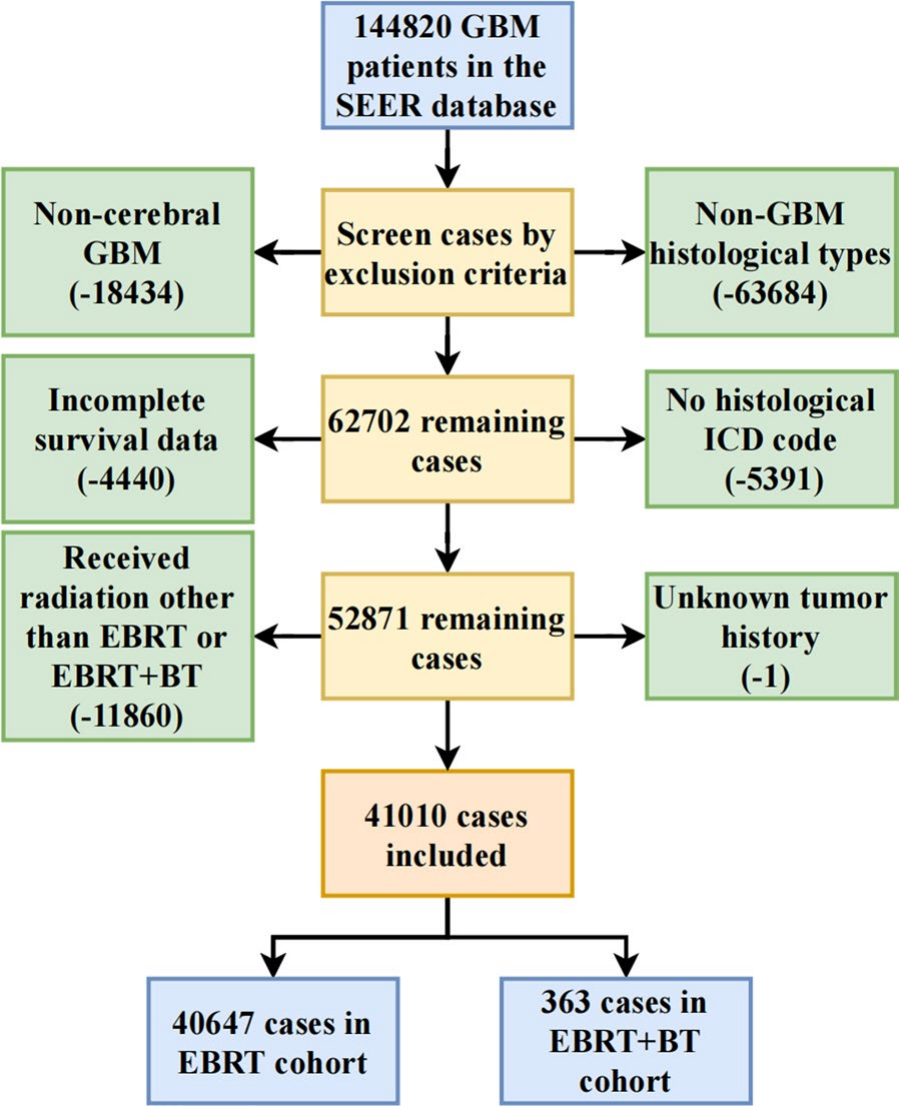
Flow chart in this study

After screening cases according to the inclusion and exclusion criteria and omitting censored cases, a total of 41,010 eligible GBM patients were included in the analysis. The flow chart is presented in Fig. 1. Of the initial cohort, 40,647 patients (99.11%) were grouped into the EBRT cohort, and 363 patients (0.89%) were grouped into the EBRT + BT cohort. As shown in Table 1, there are statistically significant differences in some demographic and clinical factors between the two groups, including age, survival time, survival events, diagnosis years, histopathologic types, surgical methods, primary tumor sites, tumor size, other malignance histories, chemotherapy (all *p* < 0.05). 36,732 patients died in the EBRT group and 354 in the EBRT + BT group. After adjusting for competing death, the number of deaths was 30,399 patients in the EBRT group and 314 patients in the EBRT + BT group.

**Table 1:** Clinical characteristics of included patients

Variables	Total (n = 41,010)	EBRT (n = 40,647)	EBRT + BT (n = 363)	*p*
Survival.months, median (Q1, Q3)	10 (5, 17)	10 (5, 17)	15 (9, 23.5)	< 0.001
Outcome, n (%)				< 0.001
Death	37,086 (90.4)	36,732 (90.4)	354 (97.5)	
Live	3924 (9.6)	3915 (9.6)	9 (2.5)	
Outcome classification, n (%)				< 0.001
Death from glioma	30,713 (74.9)	30,399 (74.8)	314 (86.5)	
Death from others	6373 (15.5)	6333 (15.6)	40 (11)	
Live	3924 (9.6)	3915 (9.6)	9 (2.5)	
Age, median (Q1, Q3)	61 (52, 70)	61 (52, 70)	55 (45, 63)	< 0.001
Age category, n (%)				< 0.001
~ 50	8589 (20.9)	8459 (20.8)	130 (35.8)	
51 ~ 60	10,756 (26.2)	10,651 (26.2)	105 (28.9)	
61 ~ 70	12,130 (29.6)	12,039 (29.6)	91 (25.1)	
71 ~	9535 (23.3)	9498 (23.4)	37 (10.2)	
Sex, n (%)				0.289
Female	16,677 (40.7)	16,519 (40.6)	158 (43.5)	
Male	24,333 (59.3)	24,128 (59.4)	205 (56.5)	
Race, n (%)				0.96
Black	2146 (5.2)	2126 (5.2)	20 (5.5)	
Others	1857 (4.5)	1840 (4.5)	17 (4.7)	
White	37,007 (90.2)	36,681 (90.2)	326 (89.8)	
Marital, n (%)				0.365
Divorced/separated	3458 (8.4)	3429 (8.4)	29 (8)	
Married	27,925 (68.1)	27,673 (68.1)	252 (69.4)	
Single/unmarried	5193 (12.7)	5141 (12.6)	52 (14.3)	
Widowed/others	4434 (10.8)	4404 (10.8)	30 (8.3)	
Diagnosis, n (%)				< 0.001
1975 ~ 2000	11,964 (29.2)	11,710 (28.8)	254 (70)	
2001 ~ 2005	7598 (18.5)	7525 (18.5)	73 (20.1)	
2006 ~ 2010	8971 (21.9)	8945 (22)	26 (7.2)	
2011 ~ 2016	12,477 (30.4)	12,467 (30.7)	10 (2.8)	
Histological type, n (%)				0.026
GBM	39,669 (96.7)	39,320 (96.7)	349 (96.1)	
gcGBM	428 (1)	419 (1)	9 (2.5)	
Gliosarcoma	913 (2.2)	908 (2.2)	5 (1.4)	
Surgery, n (%)				< 0.001
No	5154 (12.6)	5136 (12.6)	18 (5)	
Unknown	9197 (22.4)	8986 (22.1)	211 (58.1)	
Yes	26,659 (65)	26,525 (65.3)	134 (36.9)	
Surgery method, n (%)				< 0.001
Biopsy/Local.excision	5953 (14.5)	5930 (14.6)	23 (6.3)	
Gross total resection	10,994 (26.8)	10,924 (26.9)	70 (19.3)	
No surgery	5154 (12.6)	5136 (12.6)	18 (5)	
Unknown	9460 (23.1)	9249 (22.8)	211 (58.1)	
Subtotal resection	9449 (23)	9408 (23.1)	41 (11.3)	
Chemotherapy, n (%)				< 0.001
No	13,167 (32.1)	12,990 (32)	177 (48.8)	
Yes	27,843 (67.9)	27,657 (68)	186 (51.2)	
Tumor size, n (%)				< 0.001
Size ≤ 4 cm	8461 (20.6)	8445 (20.8)	16 (4.4)	
Size > 4 cm	10,894 (26.6)	10,877 (26.8)	17 (4.7)	
Unknown	21,655 (52.8)	21,325 (52.5)	330 (90.9)	
Tumor history, n (%)				0.001
No	36,267 (88.4)	35,926 (88.4)	341 (93.9)	
Yes	4743 (11.6)	4721 (11.6)	22 (6.1)	
Primary site, n (%)				0.992
Frontal	10,792 (26.3)	10,695 (26.3)	97 (26.7)	
Occipital	1851 (4.5)	1836 (4.5)	15 (4.1)	
Others	10,906 (26.6)	10,808 (26.6)	98 (27)	
Parietal	7111 (17.3)	7047 (17.3)	64 (17.6)	
Temporal	10,350 (25.2)	10,261 (25.2)	89 (24.5)	

### Survival analysis for all patients by Kaplan–Meier analysis and COX regression

**Fig. 2:**
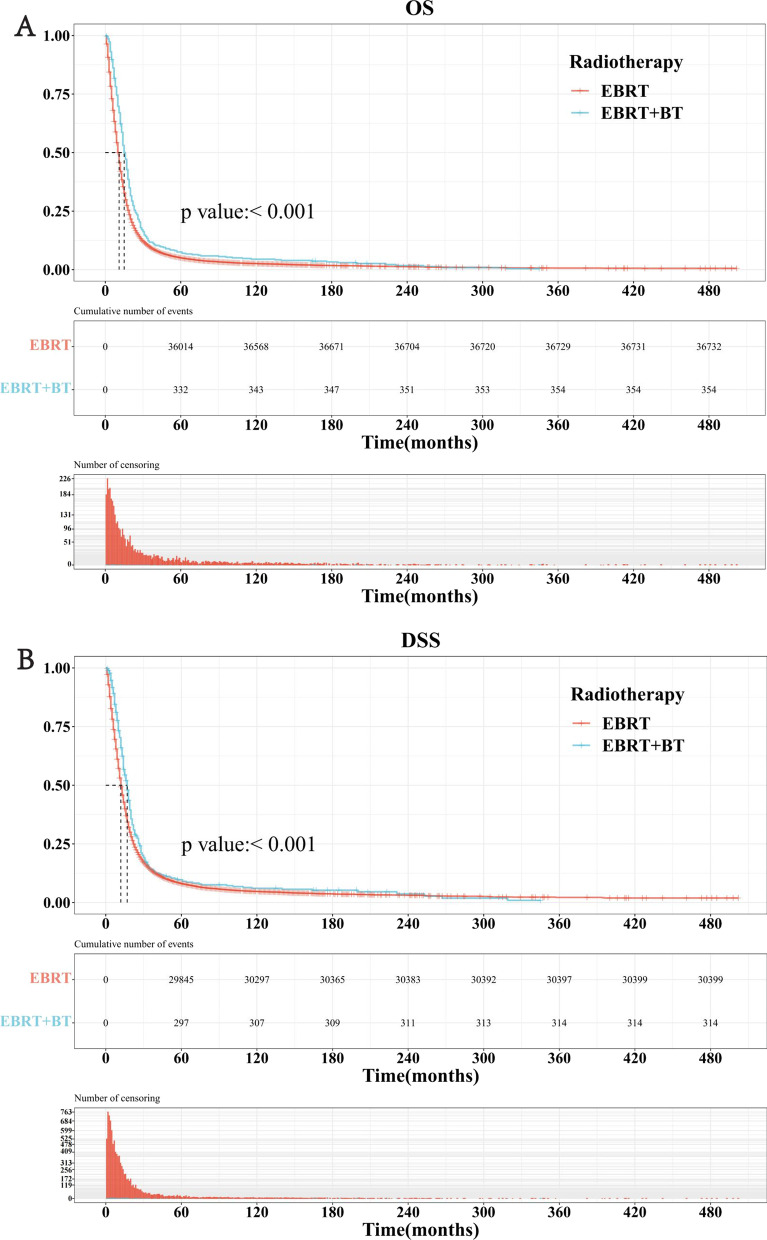
The Kaplan–Meier analysis of the EBRT and EBRT + BT groups. **A** The Kaplan–Meier survival curve for OS of the two groups. **B** The Kaplan–Meier survival curve for DSS of the two groups

The prognosis of patients in the EBRT group was significantly worse than those in the EBRT + BT group. The median survival time of OS in the EBRT + BT group (15 months, 95%CI [14, 17] months) was significantly longer than that of the EBRT group (10 months, 95%CI [10, 11] months). Likewise, the median survival time of DSS in the EBRT + BT group (17 months, 95%CI [15, 18] months) was significantly longer than that of the EBRT group (12 months, 95%CI [12, 12] months), as shown in Fig. 2A, B.

**Fig. 3:**
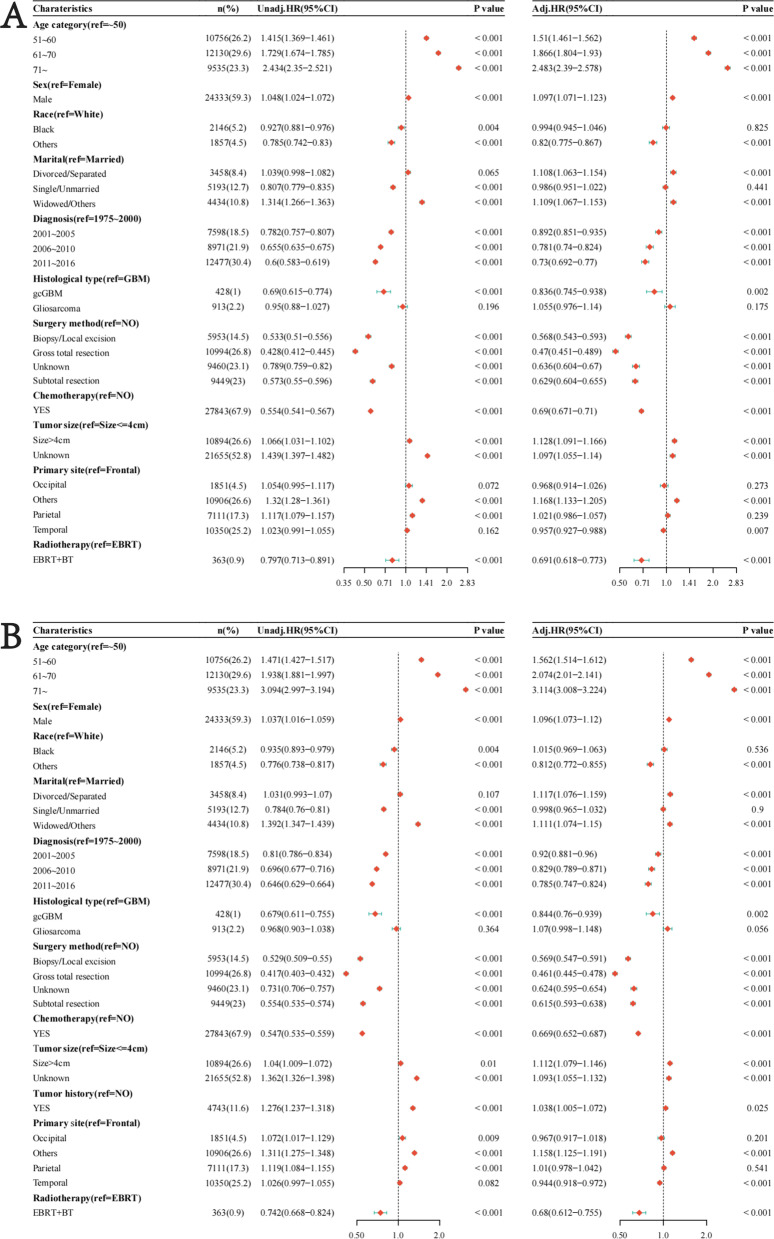
Univariate and multivariate analysis between the two groups by Cox regression. **A** Univariate and multivariate analysis with OS as the time variable. **B** Univariate and multivariate analysis with DSS as the time variable

In the univariate analysis of OS, the following factors were associated with a worse prognosis: advanced age, male, widowed or another marital status, tumor size > 4 cm, having a history of another tumor ahead, parietal or other sites located primary tumors. The factors associated with a preferable prognosis include black or other race, single or unmarried marital status, diagnosis years, histological type of gcGBM, surgery, and chemotherapy. It should be noted that the treatment of EBRT + BT was more beneficial to survival than the treatment of EBRT alone. In the multivariate analysis of OS, the following factors independently contributed to the worse prognosis: advanced age, male, divorced or separated marital status, widowed or another marital status, tumor size > 4 cm, having a prior tumor history, the primary tumor is located in other sites. As for the tumor of other sites, the definition of the “other sites” was unclear, so this result did not have much practical meaning. Of note, EBRT + BT (HR = 0.68, 95% CI [0.61, 0.75], *p* < 0.001) had more independent prognostic protection than EBRT alone (Fig. 3A). Analogous conclusions are shown for DSS in Fig. 3B.

### Univariate and multivariate analysis by competing risk model before PSM

**Fig. 4:**
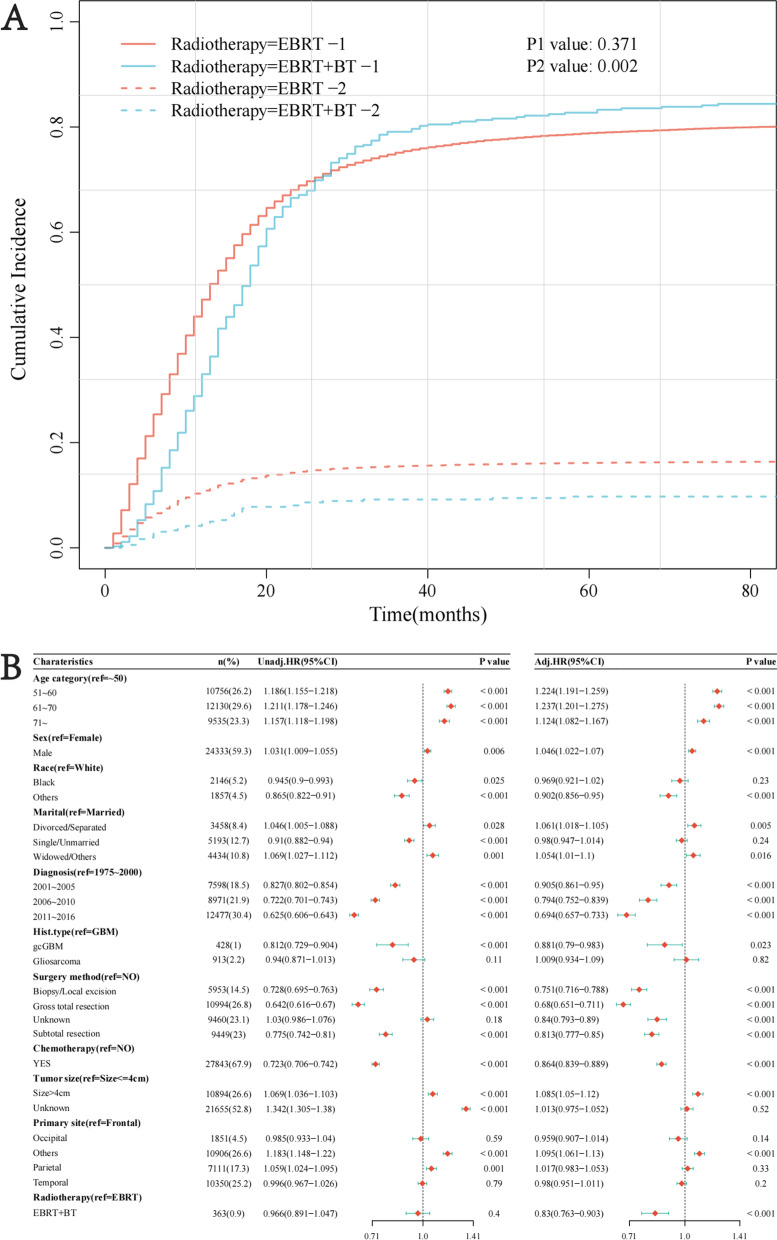
The Nelson–Aslen cumulative hazard curves of the two radiotherapies and univariate and multivariate analysis by competing risk model before PSM. **A** The cumulative hazard curve curves are constructed according to the cumulative incidence function. “1” represents that the outcome event is GBM specific death, and “2” means that the outcome event is a competitive death. **B** Univariate analysis shows no significant prognostic difference between EBRT and EBRT + BT; multivariate analysis indicates that EBRT + BT contributes to a more favorable prognosis independently

The Nelson-Aslen cumulative hazard curves for the two treatments were plotted, and Gray's test was conducted to identify the difference (Fig. 4A). It was found that there was no difference in the cumulative hazard between the two treatment groups when GBM-specific death was used as the event of interest (*p* = 0.37). When competitive death was used as the event of interest, the cumulative hazard between the two groups was statistically different (*p* = 0.002), and patients of the EBRT group had higher cumulative risks (Fig. 4B).

The Fine-Gray test was used to conduct multivariate analyses. We found that EBRT + BT was better than EBRT alone in independently improving the prognosis (HR = 0.83, 95% CI [0.76, 0.90], *p* < 0.001). In addition, advanced age, male, specific marital status, and tumor size larger than 4 cm were independently associated with poorer prognosis. Other races, diagnosis years, surgery history, and chemotherapy history could be considered independent protective factors of GBM-specific death.

### Subgroup analysis and interaction test before PSM

**Fig. 5:**
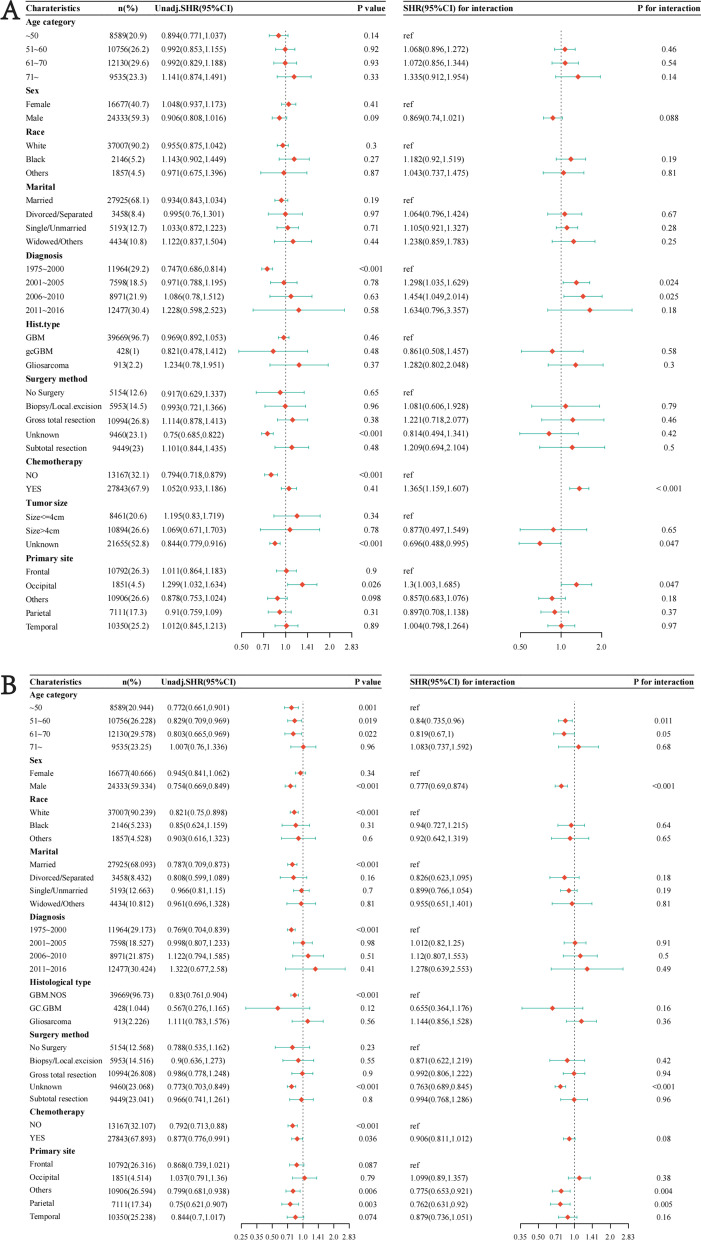
Subgroup of EBRT or EBRT + BT analysis and interaction test before PSM. **A** The association between clinical features and prognosis in only the EBRT group. **B** The association between clinical features and prognosis in EBRT + BT group. Unadj is short for Unadjusted. *SHR* sub-distribution hazard ratio

We conducted interaction tests in each subgroup, and the Log likelihood ratio test was used to test significance. Figure 5A shows that EBRT + BT has more pronounced effects in patients from 51 to 60 years old (SHR = 0.84; 95%CI [0.735, 0.96], *p* = 0.011), male patients (SHR = 0.77; 95%CI [0.69, 0.87], *p* < 0.001), patients with parietal localized GBM (SHR = 0.77, 95%CI [0.65, 0.92], *p* = 0.004). As shown in Fig. 5B, the effects of EBRT is more prominent in patients diagnosed with GBM from 2001 to 2005 (SHR = 1.29; 95%CI [1.03, 1.62], *p* = 0.024), and patients diagnosed with GBM from 2006 to 2010 (SHR = 1.45; 95%CI [1.04, 2.01], *p* = 0.02), patients underwent chemotherapy (SHR = 1.36; 95%CI [1.15, 1.60], *p* < 0.001), patients with tumor size > 4 cm (SHR = 0.77; 95% CI [0.65, 0.92], *p* = 0.004), patients with occipital localized GBM (SHR = 1.30; 95%CI [1.00, 1.68], *p* = 0.047).

### Evaluation of baseline balance after PSM

**Fig. 6:**
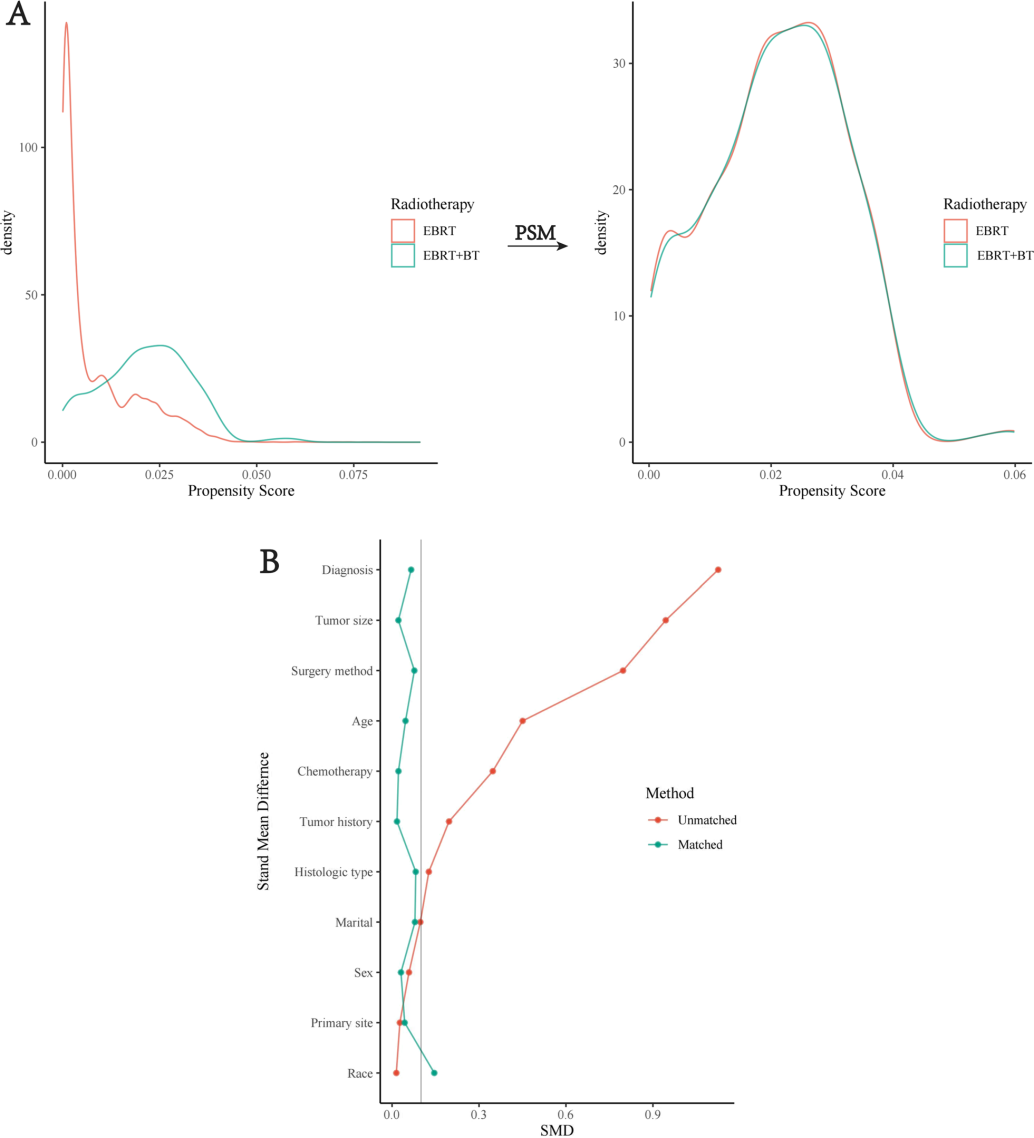
Evaluation of the balance of the clinical characteristics of the two groups after PSM. **A** Change of density map before and after PSM. **B** Change of SMD before and after PSM. *SMD* standardized mean difference

To minimize the selection bias of the two radiotherapy groups, PSM was performed. A total of 1083 patients were included, and 361 patients of the EBRT + BT group were matched with 722 patients of the EBRT group. After matching cases, the differences of these variates between the two groups become insignificant (all *p* > 0.05). All SMD of variates was lower than 0.1 except race (SMD = 0.14), as is shown in Table 2. The Kernel density plots were plotted using the density function in Fig. 6A, indicating that the propensity score is almost evenly distributed between the two groups. The love plot was plotted after PSM in Fig. 6B. The above results suggest that the essential characteristics between the two groups have reached a balance after PSM.

**Table 2:** Clinical characteristics of matched patients

Variables	Matched level	Total	EBRT	EBRT + BT	*p*	SMD
Number		1083	722	361		
Age category, n (%)	~ 50	382 (35.3)	253 (35.0)	129 (35.7)	0.914	0.046
	51 ~ 60	312 (28.8)	208 (28.8)	104 (28.8)		
	61 ~ 70	285 (26.3)	194 (26.9)	91 (25.2)		
	71 ~	104 (9.6)	67 (9.3)	37 (10.2)		
Sex, n (%)	Female	463 (42.8)	305 (42.2)	158 (43.8)	0.68	0.031
	Male	620 (57.2)	417 (57.8)	203 (56.2)		
Race, n (%)	White	997 (92.1)	673 (93.2)	324 (89.8)	0.058	0.146
	Black	53 (4.9)	33 (4.6)	20 (5.5)		
	Others	33 (3.0)	16 (2.2)	17 (4.7)		
Marital, n (%)	Married	759 (70.1)	509 (70.5)	250 (69.3)	0.678	0.079
	Divorced/separated	96 (8.9)	67 (9.3)	29 (8.0)		
	Single/unmarried	139 (12.8)	87 (12.0)	52 (14.4)		
	Widowed/others	89 (8.2)	59 (8.2)	30 (8.3)		
Diagnosis, n (%)	1975 ~ 2000	767 (70.8)	515 (71.3)	252 (69.8)	0.79	0.066
	2001 ~ 2005	211 (19.5)	138 (19.1)	73 (20.2)		
	2006 ~ 2010	70 (6.5)	44 (6.1)	26 (7.2)		
	2011 ~ 2016	35 (3.2)	25 (3.5)	10 (2.8)		
Histological type, n (%)	GBM	1053 (97.2)	704 (97.5)	349 (96.7)	0.407	0.082
	gcGBM	14 (1.3)	7 (1.0)	7 (1.9)		
	Gliosarcoma	16 (1.5)	11 (1.5)	5 (1.4)		
Surgery method, n (%)	No surgery	47 (4.3)	29 (4.0)	18 (5.0)	0.833	0.077
	Biopsy/local excision	60 (5.5)	37 (5.1)	23 (6.4)		
	Gross total resection	210 (19.4)	140 (19.4)	70 (19.4)		
	Unknown	645 (59.6)	436 (60.4)	209 (57.9)		
	Subtotal resection	121 (11.2)	80 (11.1)	41 (11.4)		
Chemotherapy, n (%)	No	536 (49.5)	360 (49.9)	176 (48.8)	0.78	0.022
	Yes	547 (50.5)	362 (50.1)	185 (51.2)		
Tumor size, n (%)	Size ≤ 4 cm	45 (4.2)	29 (4.0)	16 (4.4)	0.943	0.022
	Size > 4 cm	50 (4.6)	33 (4.6)	17 (4.7)		
	Unknown	988 (91.2)	660 (91.4)	328 (90.9)		
Tumor history, n (%)	No	1014 (93.6)	675 (93.5)	339 (93.9)	0.895	0.017
	Yes	69 (6.4)	47 (6.5)	22 (6.1)		
Primary site, n (%)	Frontal	302 (27.9)	205 (28.4)	97 (26.9)	0.978	0.044
	Occipital	45 (4.2)	30 (4.2)	15 (4.2)		
	Others	292 (27.0)	194 (26.9)	98 (27.1)		
	Parietal	191 (17.6)	128 (17.7)	63 (17.5)		
	Temporal	253 (23.4)	165 (22.9)	88 (24.4)

### Univariate and multivariate analysis by competing risk model after PSM

**Fig. 7:**
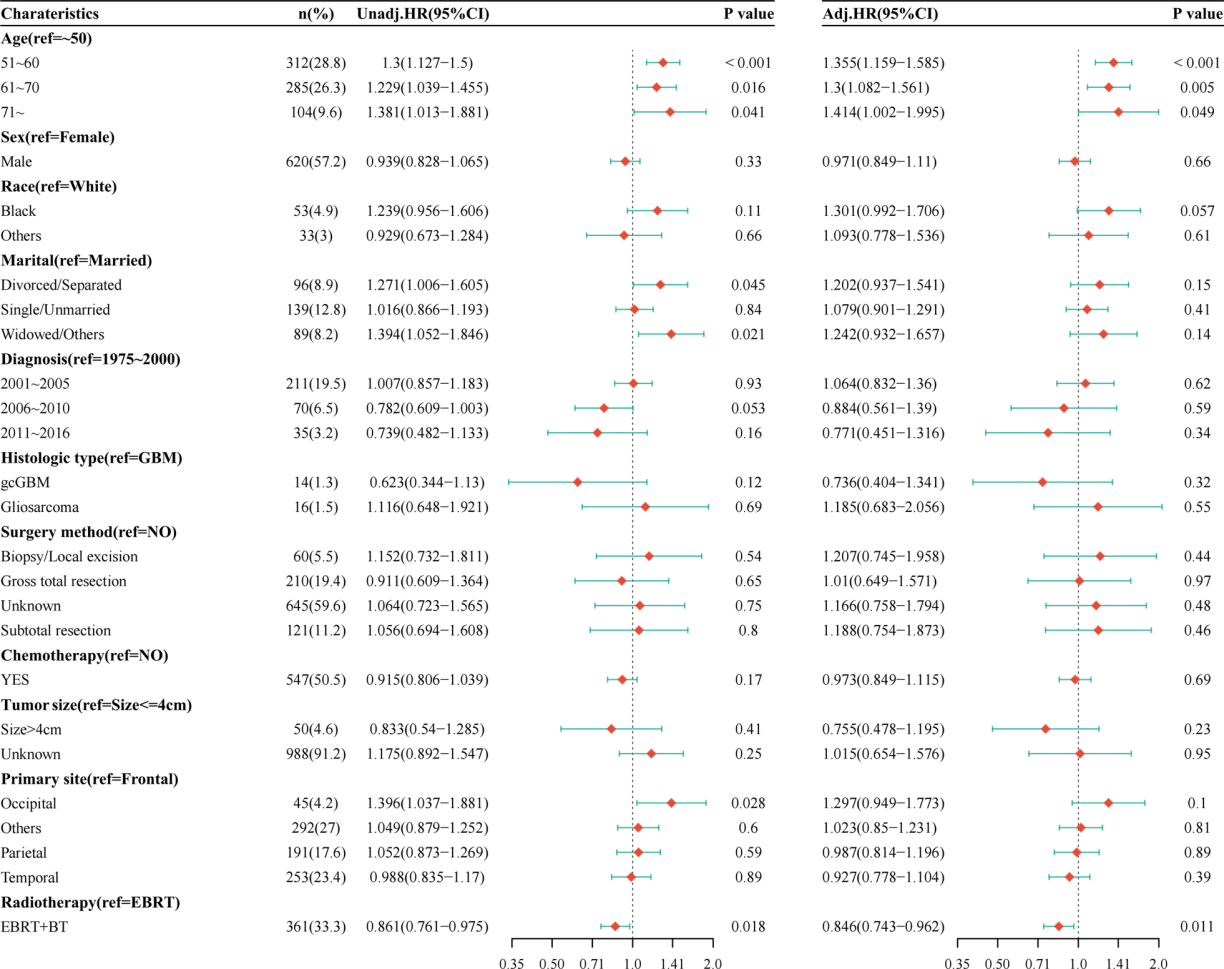
Univariate and multivariate analysis by competing risk model after PSM

In the univariate analysis after PSM, EBRT + BT decreased the risk of GBM-specific death by 14% (HR = 0.86, 95% CI [0.76, 0.97], *p* = 0.01). In multivariable analysis, we found that advanced age was independently associated with worse prognosis, and EBRT + BT (HR = 0.84, 95% CI [0.74, 0.96]. *p* = 0.011) was confirmed to independently decrease the 15% risk when compared with EBRT group. As shown in Fig. 7.

### Subgroup analysis and interaction test after PSM

**Fig. 8:**
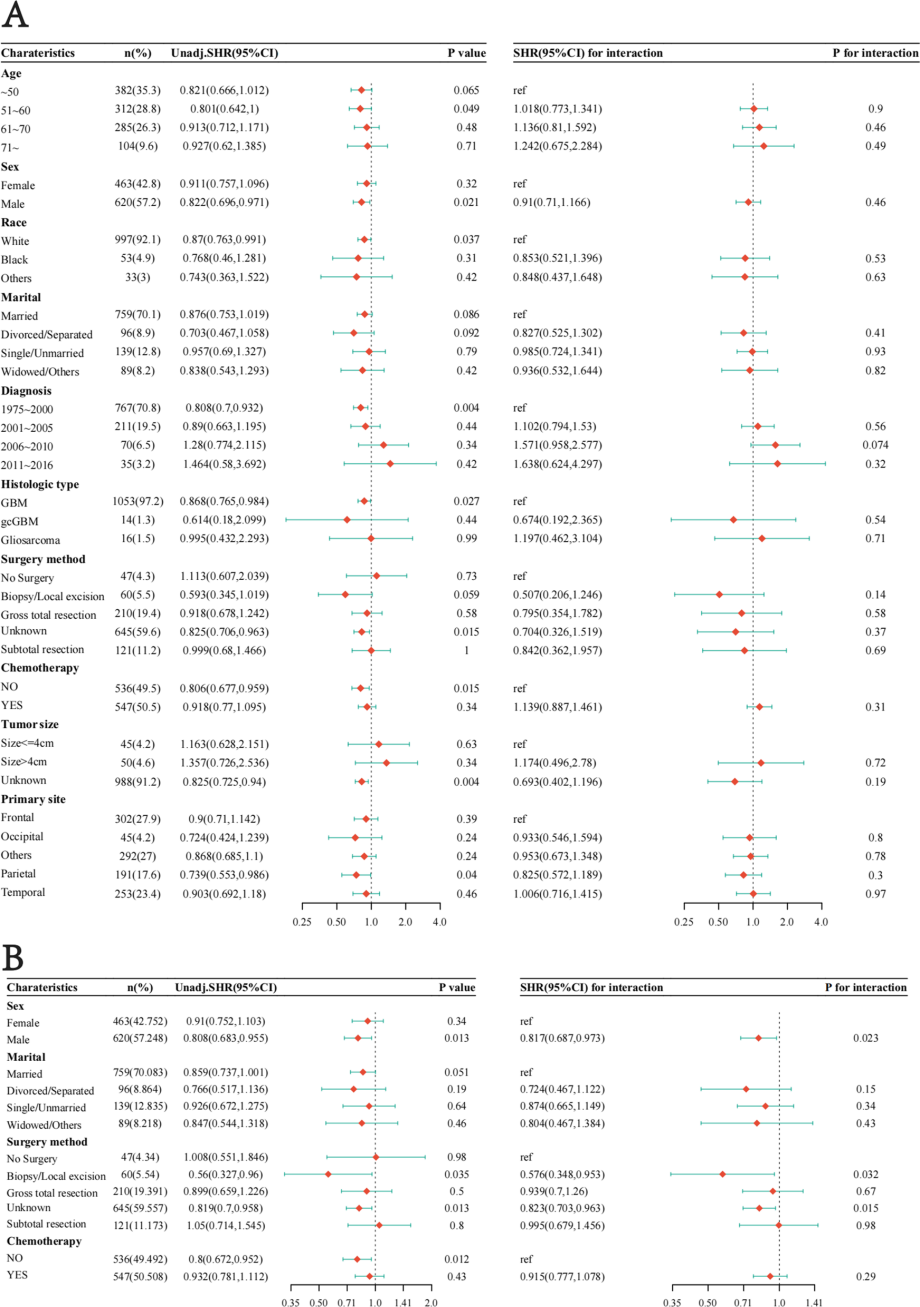
Subgroup of EBRT or EBRT + BT analysis and interaction test after PSM. **A** The association between clinical characteristics and prognosis in only the EBRT group. **B** The association between clinical features and prognosis in the EBRT + BT group

We performed the subgroup analyses and interaction tests after PSM. Figure 8A shows no interaction between EBRT and each variable. As shown in Fig. 8B, the EBRT + BT is more pronounced in treating male patients (SHR = 0.81; 95%CI [0.68, 0.97], *p* = 0.02), patients underwent biopsy or local excision (SHR = 0.57, 95%CI [0.34, 0.95], *p* = 0.03).

## Discussion

GBM accounts for more than 60% of adult brain tumors and about 3% of childhood brain tumors [4]. The current standard adjuvant EBRT for GBM is advocated [14]. BT has been fully developed under the continuous development of imaging technology. The most commonly used isotopes for BT are iodine 125 (I-125) and iridium 129 (Ir-192) [15]. Two studies were affecting the role of BT combined with EBRT in GBM treatment. One study was proposed by Laperriere et al. and included 140 patients with malignant astrocytoma [16] after randomizing patients into two postoperative treatment groups: 50 Gy EBRT plus 60 Gy BT or 50 Gy EBRT alone; it was found that the median OS of the BT and non-BT groups were 13.2 and 13.8 months, respectively, but with no significance (*p* = 0.49). Another study included 299 patients and divided the patients into two groups randomly, either 60 Gy EBRT plus carmustine or 60 Gy (40 cGy/h) BT followed by the same EBRT and carmustine, the results turned out to be that increased survival was observed in the latter group (15.7 vs. 13.5 months), but the survival advantage was not significant (*p* = 0.10) [17]. Given the above results, it may be the right choice to regard BT as a supplement to the standard treatment. The authors reported that compared with patients who received only EBRT and temozolomide treatment after surgery, the PFS of patients who received BT immediately after surgery, followed by the same EBRT and chemotherapy, had a considerable improvement [18]. Welsh et al. showed that in combination with 50 Gy (53 cGy/h) BT, standard treatments brought a 3-month more prolonged survival [19].

Through univariate and multivariate Cox analyses, we found that many factors affect the prognosis of GBM patients, such as age, marital status, gender, primary tumor location, etc. It is noteworthy that the prognosis of patients who received EBRT + BT was better than that of patients who received EBRT alone, the risk of all-cause death was reduced by 32%, and the risk of disease-specific death was decreased by 30.9%. Since OS and DSS are not equal when death is defined as the same event, the competing death exists, and results obtained from the Cox analyses are unreliable. The authoritative SEER database recorded competitive deaths. In all 41,010 patients, 15.54% patients died from competing causes. The ratio is high enough to cause considerable interference. Most of our currently established knowledge is based on studies of inpatients, and non-tumor-related and non-hospital deaths are often considered right-censored data, which can’t reflect the actual situation of a large population, so we think it is optimal to use a competing risk model to eliminate the potential bias.

Through univariate and multivariate analysis of the competing risk model, we found that aging, males, widowed, divorced, and larger tumor size (> 4 cm) are all independent risk factors that affect the survival of patients, and other colored races except blacks, the year of diagnosis, gcGBM, any surgery, and chemotherapy are all independent protective factors that improve the survival of the patient. The EBRT + BT can protect the patient from unfavorable prognosis better than the EBRT alone, and the EBRT + BT can also be regarded as an independent protective factor for the patient’s prognosis. After PSM, the matched two cohorts revealed no significant differences in the baseline. Unlike the above results, multivariate analysis after PSM shows that only age is an independent prognostic risk factor. But EBRT + BT is still an independent prognostic protective factor; that is, compared with EBRT alone, the treatment effect of EBRT + BT is better. In the subgroup analysis after PSM, we found that EBRT had universal applicability in treating GBM. At the same time, EBRT + BT was more effective in treating males with GBM who had undergone local resection.

Consistently, a study that included 273 elderly GBM patients showed that compared with patients older than 75 years, the OS of those between 65- and 74- years patients was significantly longer (9.8 ± 10.8 vs. 5.2 ± 5.2 months, *p* = 0.0004) [20]. And a meta-analysis showed an increased HR of mortality was positively associated with increasing age [21]. This may be partly due to the older patients’ brain tissues becoming more sensitive to reactive oxygen species damage and more immunosuppressed in the brain [22]. Compared with the female, the male has a higher incidence and mortality of GBM, and this difference exists between men and women of all ages, so this difference may not only be related to sex hormone levels but also may be related to gene expression and gene modification difference between the sexes. A study has revealed the gender-specific molecular subtypes of GBM, in which cell cycle and integrin signaling are the critical determinants of survival in male and female patients, respectively [23]. Marital status can also affect the prognosis of GBM. The survival benefit of marriage for prognosis is particularly prominent in men over 60 years of age, white people, or patients living in middle-income countries [24]. One possible explanation is that married patients may have better economic status, social support, and psychological comfort than unmarried or widowed patients. It has always been a consensus that tumor diameter is closely related to the prognosis of GBM patients. Tumor size > 5.4 cm was identified as a risk factor for GBM in a study for the elderly [25]. Yang Xu et al. proposed that the repression of p38 phosphorylation could promote GBM cell autophagy and apoptosis, and tumor size was inversely correlated with p38 phosphorylation and positively correlated with RND2, a key inhibitor of phosphorylation of p38 [26]. A large-scale population study of 150,631 GBM patients reported that Asian and Pacific residents were defined as having a better prognosis than whites and blacks [27]; the conclusion is similar to ours. But other population-based studies didn’t demonstrate a race-based disparity in GBM survival [28]. The gcGBM is an uncommon subtype of GBM, accounting for approximately 4% of GBM patients. Michael CJ et al. found that compared with general GBM patients, the survival time of gcGBM patients was improved (15.5 months vs. 11.7 months, *p* < 0.001) [29]. And patients with gcGBM can obtain more survival benefits from surgery and radiotherapy [30]. In our results, the prognosis of patients undergoing surgery or chemotherapy is undoubtedly better, and it is worth mentioning that patients who receive complete resection have the best prognosis. M Lacroix et al. pointed out that when the resection volume was less than 98% Of GBM volume, the patient’s median survival time was only 8.8 months. Still, when the resection volume was greater than 98% GBM volume or total resection, the patient’s median survival time was significantly increased to 13 months; therefore, complete resection as far as possible under the premise of safety should be the key to improving the prognosis of patients [31].

Our study has some limitations. Firstly, our conclusions are mainly drawn from the analysis of SEER data; although our study embraces a large sample size, there is no other external data verification. Secondly, the variables are limited, and some critical variable information is not provided or is vague in the SEER database, such as “No/Unknown,” “Others,” and “Not Available.” Therefore, results related to "Others" in this study should be treated with caution. Lastly, our retrospective study has a lower evidence power than prospective studies.

There are also some advantages to our study. First of all, our study is the first to explore the difference between the efficacy of EBRT and EBRT + BT using a large population in the SEER database, so it is innovative. Moreover, we have effectively used competing risk models and PSM to eliminate bias and make our results more credible. Finally, we analyzed the possible factors that interacted with EBRT or EBRT + BT in the subgroup analysis and found the specific population that may be more sensitive to EBRT + BT.

In conclusion, this large population-based study provided a reliable statistical analysis and found that the difference in prognosis between the two types of radiotherapy was statistically significant. The interaction variables with the radiotherapy regimens have also been identified, which may contribute to future precise radiotherapy for GBM patients.

## Data Availability

The data used to support the findings of this study are available from the corresponding author upon request.

## Abbreviations

BT: Brachytherapy
CIF: Cumulative incidence function
DSS: Disease-specific survival
EBRT: External beam radiation therapy
GBM: Glioblastoma multiforme
gcGBM: Giant cell GBM
HR: Hazard ratio
ICD-O-3: International Classification of Disease for Oncology 3rd edition
OS: Overall survival
PSM: Propensity score matching
SEER: Surveillance, epidemiology, and end results
SHR: Sub-distribution hazard ratio
SMD: Standardized mean difference
